# Supplementary material for: Circulating nucleosomes as new blood-based biomarkers for detection of colorectal cancer
Source: Clin Epigenetics. 2017 May 15;9:53. doi: 10.1186/s13148-017-0351-5 (PMC5433015; doi:10.1186/s13148-017-0351-5)
Supplement: Additional file 1: Table S1. — Absence of correlation between NuQ® assays, age, and gender. Values are expressed in Pearson correlation coefficient. (DOC 30 kb) [file 13148_2017_351_MOESM1_ESM.doc]

Table S1: Absence of correlation between NuQ® assays, age and gender

|  |  | **gender** | **age** | **smoking** |
| --- | --- | --- | --- | --- |
| **gender** | | 100% | -9% | -5% |
| **age** | | -9% | 100% | 3% |
| **smoking** | | -5% | 3% | 100% |
| **NuQ® assay** | **Nucleosomes** | 0% | -28% | -3% |
|  | **H2AK119Ub** | -5% | 23% | -3% |
|  | **H3K9Ac** | -2% | -18% | -13% |
|  | **H3K27Ac** | -5% | -16% | 2% |
